# CTPS2 regulates CTP synthetase activity by interacting with CTPS1

**DOI:** 10.26508/lsa.202403117

**Published:** 2025-09-16

**Authors:** Norbert Minet, Benoît Heid-Picard, Vanessa Masson, Damarys Loew, Emmanuel Martin, Sylvain Latour

**Affiliations:** 1 Laboratory of Lymphocyte Activation and Susceptibility to EBV infection, Inserm UMR 1163, Institut Imagine, Paris, France; 2 Université de Paris, Paris, France; 3 Institut Curie , PSL Research University, Centre de Recherche, CurieCoreTech Mass Spectrometry Proteomics, Paris, France

## Abstract

CTP synthetases (CTPS) 1 and 2 are responsible for intracellular de novo production of the CTP nucleotide required for DNA replication and cell proliferation. CTPS1 and CTPS2 are co-expressed in most of tissues and share a high structural homology. They form homotetramers that are enzymatically active and aggregate into large intracellular storage filament-like structures termed as cytoophidia. Herein, we found that CTPS1 and CTPS2 co-localized in cytoophidia when co-expressed and CTPS2-containing filaments are dependent on CTPS1 expression. Cytoophidia were not necessary for proliferation because CTPS1^H355A^ and CTPS2^H355A^ mutants that are unable to form cytoophidia could sustain normal cell proliferation. CTPS1 and CTPS2 were found to directly interact together independent of polymerization and formation of cytoophidia. When associated with CTPS2, CTPS1 enzymatic activity was decreased and more sensitive to CTP/product negative feedback, suggesting that the presence of CTPS2 modulates CTPS activity. Therefore, our results demonstrate that CTPS1 and CTPS2 do not only function independently, but also associate and form complexes in the absence of polymerization, suggesting the possibility that they directly regulate each other through heterotetramerization.

## Abstract (precis)

The human CTP synthetases 1 and 2 associate together and co-localize in large filament–cytoophidium structures. CTPS1 and CTPS2 association results in enzymatic modulation independently of cytoophidia revealing a new regulation mechanism of CTP synthetase activity.

## Introduction

In humans, the final steps of de novo CTP production rest on the activity of the cytidine triphosphate synthetases (CTPS) that are encoded by the two genes *CTPS1* and *CTPS2*. Cellular CTP levels appear to be tightly controlled, leading to low levels in normal tissues compared with other key nucleotides. In turn, this restricts CTP-dependent metabolic processes, such as RNA, DNA, phospholipid synthesis, and protein sialylation (Kennedy & Weiss, 1956; Roseman, 1962). As some of these processes are essential for cell proliferation, increased CTP synthetase activity levels have been observed in cancer (van den Berg et al, 1991, 1994, 1993) and activated immune cells (Verschuur et al, 1999), making it an attractive therapeutic target in cancer and lymphoproliferative disease treatment. Recently, there was a renewed interest in generating inhibitors to CTPS activity that selectively target CTPS1 for therapeutic use, to control immune responses (Martin et al, 2014, 2020; Lynch et al, 2021), cancer cell proliferation (Sun et al, 2022), and viral replication (Rao et al, 2021).

The first evidence of the importance of CTPS1 in vivo was given by the identification of a hypomorphic mutation in *CTPS1* within a cohort of patients with severe immunodeficiency (Martin et al, 2014, 2020). More recently, analyses of mice and cellular models in which *CTPS1* and/or *CTPS2* have been inactivated have shown that CTPS1 and CTPS2 are not equivalent (Minet et al, 2023; Soudais et al, 2024). *CTPS1* but not *CTPS2* is an essential gene for embryonic development and is notably required for growth of tissues with high renewal and proliferation rates such as intestinal epithelium, erythroid and thymic lineages, and activated B and T lymphocytes (Soudais et al, 2024). These observations support that CTPS1 is the main contributor to CTPS activity and the de novo production of CTP (Martin et al, 2014, 2020; Minet et al, 2023).

The differences between CTPS1 and CTPS2 could be explained on the one hand by a higher intrinsic CTPS activity of CTPS1 (Minet et al, 2023) and on the other hand by different regulatory mechanisms (Lynch et al, 2021). The active form of both enzymes is a tetramer, which is stabilized by nucleotide binding, and further switches between active and inactive conformations depending on substrates (ATP, UTP) and product (CTP) availability. Interestingly, CTPS1 possesses a single inhibitory CTP binding site near the UTP binding site, CTPS2 possesses two inhibitory binding sites, respectively overlapping partially the UTP and ATP binding sites (Lynch et al, 2021). However, the role of this additional CTP binding site in CTPS2 has not been addressed and the relative half-maximal inhibitory concentration (IC50) for CTP for each enzyme at physiological concentrations has not been determined, although CTP was found to be more potent in inhibiting CTPS2 than CTPS1 in vitro in saturating conditions of UTP and ATP (Minet et al, 2023).

Both CTPS1 and CTPS2 tetramers are able to form polymers and large intracellular filament-shaped membraneless organelles termed cytoophidia that both play an important role in their regulation. Polymers reported as thin filaments are detected by cryo-EM (Lynch et al, 2017), whereas cytoophidia are observed by light microscopy (Liu, 2010; Carcamo et al, 2011). They are different structures not related with no clear evidence today that polymers are incorporated into cytoophidia. Cytoophidia represent storage forms of inactive forms of the enzyme sequestered on intermediate cytokeratin filaments in response to nutrient stress via a methyl-dependent modification (Lin et al, 2018; Chakraborty et al, 2020). In vitro, formation of human CTPS1 polymers requires active and substrate-bound tetramers, whereas conformational changes induced by the binding of the product CTP lead to the dissociation of these structures (Lynch et al, 2017). In contrast to CTPS1, both active and inactive tetramers of CTPS2 can form polymers, and available data suggest that these tetramers can dynamically switch between the active and inactive conformation inside the polymer without prior dissociation (Lynch & Kollman, 2020). This may allow them to also modulate the conformation and therefore the activity of adjacent tetramers inside the structure through a mechanism known as conformational spread (Lynch & Kollman, 2020). In this case, it has been hypothesized that minute changes in nucleotide levels could be able to sensitively modify the activity of most of the CTPS2 pool with a responsivity akin to an on/off switch.

CTP synthases and their capacity to form polymers and cytoophidia are highly conserved across evolution. CTPS1 and CTPS2 are viewed as homotetramers and have been studied individually most of the time. As CTPS1 and CTPS2 are co-expressed in some tissues and cell types, they might interact together with functional consequences. Herein, using CTPS1- and/or CTPS2-deficient cell lines, we found that CTPS1 and CTPS2 interact together independently of cytoophidium formation and this interaction regulates CTPS1 activity and increases the inhibitory feedback loop by the CTP product, revealing a novel layer of CTP synthase regulation. Interestingly, we also showed that CTPS1- and/or CTPS2-dependent cell proliferation occurs in the absence of cytoophidium formation and thus does not depend on cytoophidium formation.

## Results

### CTPS1 and CTPS2 filament formation requirements are different

The capacity of CTPS enzymes to aggregate into cytoophidia is considered to be an important regulatory mechanism of CTPS activity. CTPS1 cytoophidium formation has been shown to be triggered by a treatment with DON or 3-DU, two CTPS competitive inhibitors of the glutamine and UTP substrates, respectively, and disrupted by high CTP concentrations (Carcamo et al, 2011; Chang et al, 2015).

**Figure 1.**
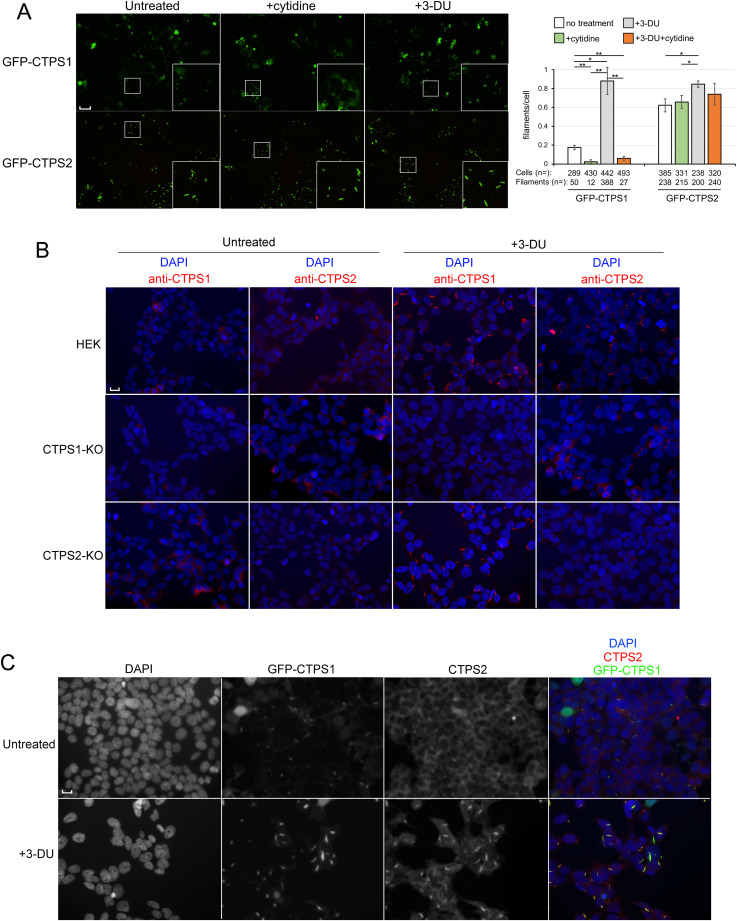
CTPS1 and CTPS2 co-localize in cytoophidia when co-expressed. **(A)** CTPS1+2-KO stably reconstituted with GFP-CTPS1 or GFP-CTPS2 were analyzed for GFP-positive cytoophidia-shaped staining. Cells were nontreated (untreated) or cultured in the presence of 3-deazauridine (3-DU) (40 μM) or cytidine (200 μM). Magnification 20x, scale bar: 100 μm, IncuCyte images, except on the bottom right of each field at magnification 60x. Right bar graphs correspond to the filament–cytoophidium/cell quantification with indicated numbers of cells and filaments–cytoophidia counted (n=) in three independent fields/images for each condition. Images of the condition 3DU+cytidine are not shown. **(B)** Endogenous cytoophidia of CTPS1 and CTPS2 in WT HEK (HEK), CTPS1-KO, and CTPS2-KO HEK cells treated or not (untreated) with 40 μM 3-DU detected by immunostaining with anti-CTPS1 (in red) or anti-CTPS2 (in red) with DAPI in blue. **(C)** Endogenous CTPS2 (in red) and GFP-CTPS1 cytoophidia in CTPS1-KO cells reconstituted with GFP-CTPS1. CTPS2 filament detected by anti-CTPS2 antibody. Cells were treated or not with 3-DU. **(B, C)** 40x magnification, scale bar: 10 μm. **(A, B)** Data from one representative experiment of three independent experiments in (A) and two in (B). **(A)** Data as means ± SD. Mann–Whitney statistical tests, *P < 0.05 and **P < 0.01. n = is indicated in the panel.

**Figure S1.**
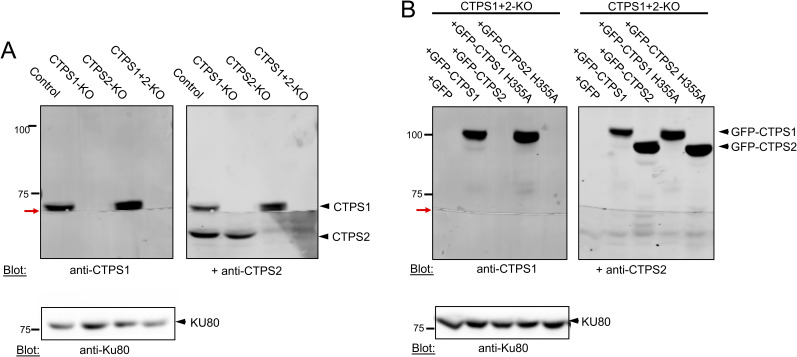
Expression of CTPS1, CTPS2, GFP-CTPS1, and GFP-CTPS2 in the different stable cell lines derived from HEK cells. **(A, B)** CTPS1 and CTPS2 expression by Western blotting in cell lysates. Membranes were first incubated with anti-CTPS1 and then with anti-CTPS2, and staining was revealed by LI-COR. Anti-KU80 blot (lower panel) as a loading control. **(A)** Lysates from WT HEK (control), CTPS1-deficient (CTPS1-KO), CTPS2-deficient (CTPS2-KO), or CTPS1+CTPS2-deficient (CTPS1+2-KO) HEK cells. **(B)** Lysates from CTPS1+2-KO HEK stable cell lines that expressed GFP alone (GFP), GFP-CTPS1, GFP-CTPS1 H355A, GFP-CTPS2, or GFP-CTPS2 H355A. Of note, the two membranes were cut accidentally in this experiment as indicated by the red arrows.

We took advantage of our previously described CTPS1- and CTPS2-KO HEK (CTPS1+2-KO) cell model stably expressing N-terminal GFP-tagged CTPS1 or CTPS2 (Minet et al, 2023) to study CTPS cytoophidium formation because the GFP allowed an easy visualization of CTPS cytoophidia (Figs 1 and S1A). We first noticed that GFP-CTPS1 and GFP-CTPS2 adopted a spontaneous cytoophidia-shaped localization when stably expressed in these cells (Figs 1A and S1B). This effect could be partially attributed to GFP dimerization (Chang et al, 2018). Interestingly, there were more cells containing GFP-CTPS2 cytoophidia than cells with GFP-CTPS1 cytoophidia. Indeed, a large proportion (62%) of cells expressing GFP-CTPS2 contained at least one GFP-CTPS2 cytoophidia, whereas a significant proportion (83%) of cells expressing GFP-CTPS1 have a diffuse cytoplasmic staining without filament-shaped staining. As expected, a treatment with the uridine analog 3-deazauridine (3-DU) markedly increased GFP-CTPS1 cytoophidium formation (Chang et al, 2015). Conversely, supplementation with cytidine, which increases intracellular CTP concentration through the salvage pathway, reduced GFP-CTPS1 cytoophidium formation. This might be attributed to a negative feedback mechanism, where CTP in excess binds to CTPS1, shifting CTPS1 tetramers into an inactive conformation (Lynch et al, 2017) that, in turn, disrupts cytoophidium formation. In striking contrast, no perceptible changes in GFP-CTPS2 cytoophidium formation were observed after cytidine treatment, whereas 3-DU treatment induced a moderate increase. This could be explained by the recent findings that substrate-bound (UTP-bound) and product-bound (CTP-bound) forms of CTPS2 tetramer both form polymers (Lynch & Kollman, 2020).

**Figure S2.**
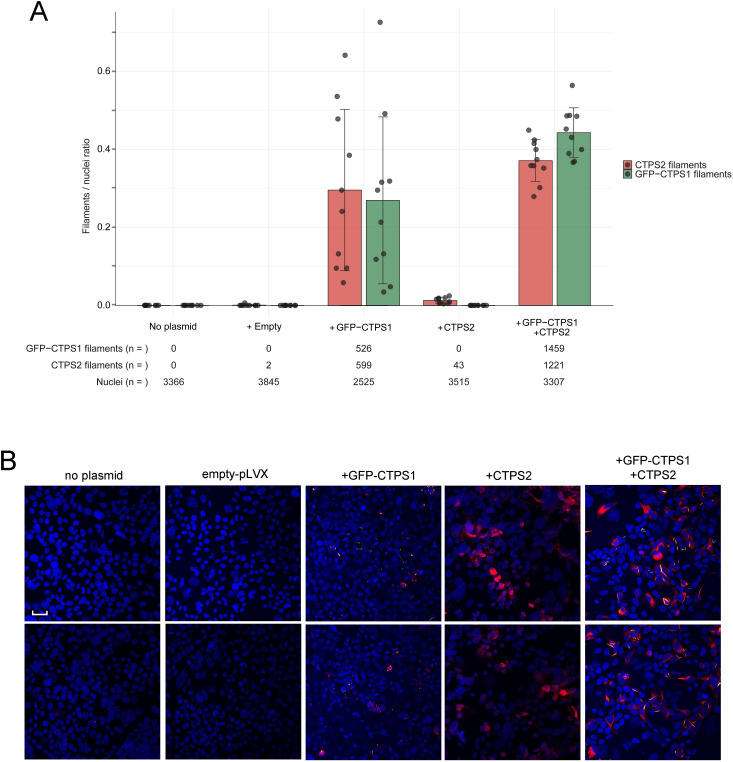
Overexpression of CTPS2 induces no cytoophidia or few very rare cytoophidia that are strongly formed in the presence of GFP-CTPS1. **(A, B)** CTPS1-KO HEK cells were transiently co-transfected with no plasmid, an empty plasmid (Empty), or plasmids coding GFP-CTPS1, CTPS2, or both GFP-CTPS1 and CTPS2 plasmids. CTPS2 was detected with a rabbit anti-CTPS2 antibody revealed by Alexa Fluor 647–coupled anti-rabbit antibody, whereas GFP-CTPS1 was directly detected through the GFP green fluorescence. **(A)** Bar graphs correspond to the filament/cell quantification from images, and total numbers of cells and cytoophidia–filaments were counted in independent fields/images for each condition indicated below. Each field/image corresponds to one point in the bar graphs with a respective mean of 337, 384, 252, 352, and 331 cells/field analyzed for the conditions no plasmid, empty plasmid, GFP-CTPS1, CTPS2, and GFP-CTPS1+CTPS2. Nuclei/cells were stained with DAPI. **(B)** Two representative fields of each condition stained with the rabbit anti-CTPS2 antibody revealed by Alexa Fluor 647–coupled anti-rabbit antibody (red) showing the overexpression of cytoplasmic CTPS2 (+CTPS2) and CTPS2 cytoophidia (+GFP-CTPS1, +GFP-CTPS1+CTPS2). Magnification 40x, Scale bar: 25 μm.

We next assessed the presence of endogenous CTPS cytoophidia in WT HEK (Fig 1B). In contrast to GFP-CTPS1 and GFP-CTPS2 cytoophidia, CTPS1 or CTPS2 cytoophidia were only observed upon 3-DU treatment. However, we cannot exclude that we missed small and/or thin CTPS1/2 cytoophidia under the threshold of detection of our staining method. Indeed, it has been proposed that cytoophidia that are large filament structures arise from the aggregation of thin preexisting filaments formed of polymers resulting from the ordered association of CTPS1/2 tetramers (Liu, 2010; Gou et al, 2014). Intriguingly, in CTPS1-KO HEK cells, CTPS2 cytoophidia were absent or very rare after treatment with 3-DU, whereas in CTPS2-KO cells, CTPS1 cytoophidia were still present, suggesting that CTPS1 is required for the formation of detectable endogenous CTPS2 cytoophidia. Expressing GFP-CTPS1 in CTPS1-KO HEK cells restored cytoophidia-shaped CTPS2 staining that perfectly co-localized with GFP-CTPS1, even in the absence of 3-DU induction (Fig 1C). Formation of CTPS2 cytoophidia could also necessitate a threshold expression level of CTPS2. However, the forced or overexpression of CTPS2 in CTPS1-KO cells resulted in very rare CTPS2 cytoophidia that are markedly induced in the presence of GFP-CTPS1 (Fig S2A and B). Thus, the absence of CTPS2 cytoophidia did not likely result in an insufficiency of CTPS2 expression. However, these data suggest that CTPS1 and CTPS2, when are co-expressed in cells, form cytoophidia together and that CTPS2 cytoophidium formation is dependent on CTPS1.

### CTPS1 and CTPS2 interaction is independent of filament formation

**Figure 2.**
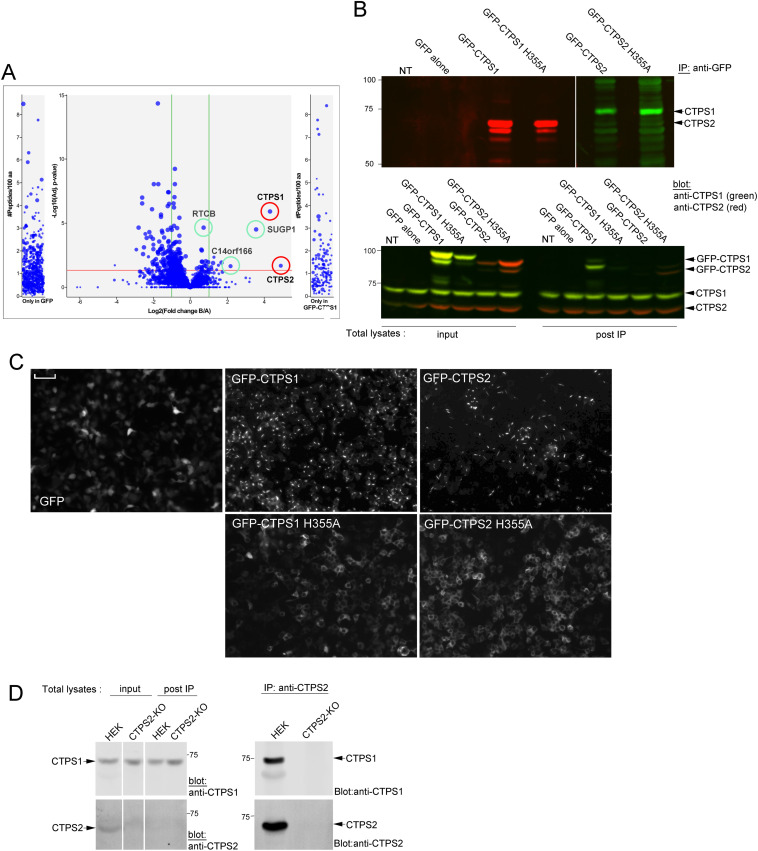
Association between CTPS1 and CTPS2 does not require cytoophidium formation. **(A)** Volcano plot analysis of interacting proteins with GFP-CTPS1 identified by quantitative mass spectrometry (compared with GFP alone). CTPS1 and CTPS2 are in red circles, and significant other proteins are in green circles (red line indicating adjusted *P* = 0.05). Data from four independent analyses of independent cell samples in each experiment. **(B)** Co-immunoprecipitation experiments from lysates of CTPS1-KO cells expressing GFP alone, GFP-CTPS1, GFP-CTPS2, GFP-CTPS1^H355A^, or GFP-CTPS2^H355A^. In the upper panels, immunoprecipitations with beads coupled to anti-GFP antibodies followed by Western blotting with anti-CTPS1 or CTPS2. In the lower panels, total lysates before IP (input) or after IP (post-IP). Of note, some signal with GFP-CTPS1 remains in the post-IP (lower panel) indicating that anti-GFP beads did not fully deplete GFP-CTPS1 contained in the lysate. **(A, C)** GFP-positive cytoophidia in the corresponding cell lines used in panel (A). Images taken at the same time as the co-immunoprecipitation experiment (magnification 20x, scale bar: 33 μm). **(D)** Co-immunoprecipitation experiments from lysates of WT HEK cells. In the upper panels, immunoprecipitations with anti-CTPS2 followed by Western blotting with anti-CTPS1 or anti-CTPS2. In the lower panels, total lysates before IP (input) or after IP (post-IP). **(B, C, D)** Data from one representative experiment of three independent experiments in (B), three in (C) except for the GFP-H355A tested one time, and three in (D).

**Figure S3.**
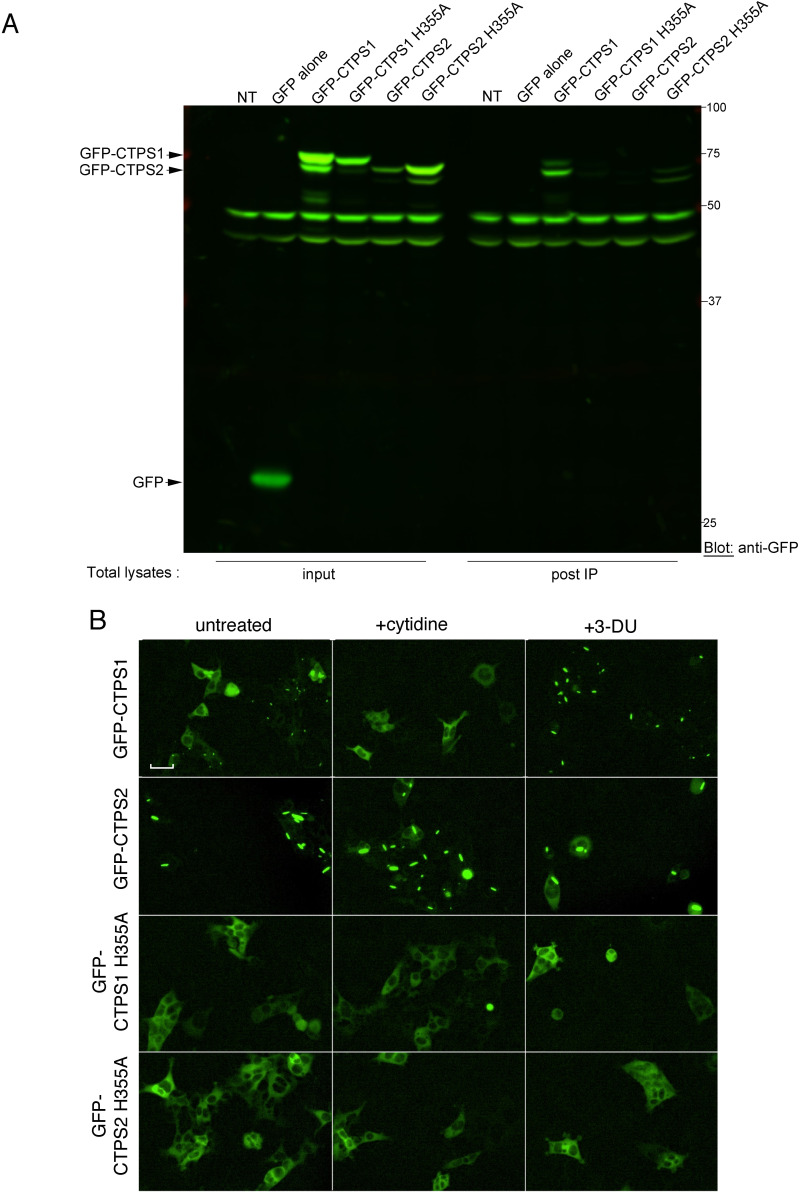
Control Western blot of Fig 2B, and GFP-CTPS1^H355A^ and GFP-CTPS2^H355A^ mutants fail to form cytoophidia. **(A)** Total lysates before IP (input) or after IP (post-IP) immunoblotted with anti-GFP antibody showing that different GFP-tagged constructs are expressed including the GFP alone. **(B)** CTPS1+2-KO HEK cells stably reconstituted with GFP-CTPS1, GFP-CTPS2, GFP-H355A-CTPS1, or GFP-H355A-CTPS2 were analyzed for GFP-positive cytoophidia. Cells were nontreated (untreated) or treated with 40 μM 3-deazauridine (3-DU) or 200 μM cytidine. Data from one representative experiment of two independent experiments except for the treatment with cytidine that was tested one time. Magnification 60x, scale bar: 20 μm, IncuCyte images.

**Figure S4.**
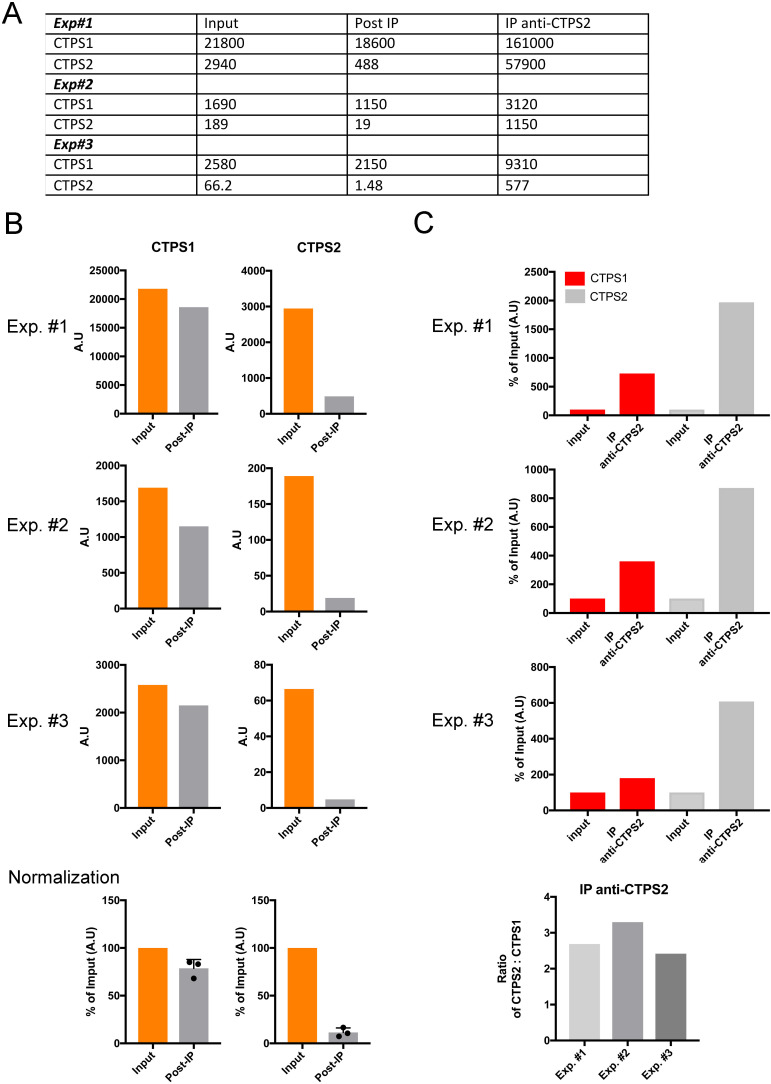
Quantification of CTPS1 and CTPS2 in immune complexes and lysates after immunoprecipitation with anti-CTPS2 in WT HEK cells. **(A, B, C)** Three experiments (Exp#1, Exp#2, and Exp#3) analyzed same as in Fig 2D, which corresponds to Exp#3. **(A, B, C)** Table of values of quantifications of bands corresponding to CTPS1 and CTPS2 from Western blots used in (B, C). Quantifications in arbitrary units (A.U.). **(B)** Comparison of CTPS1 and CTPS1 in the “input” and after immunoprecipitation “Post-IP” in the three experiments indicating that 80–95% of the CTPS2 and 17–27% of the CTPS1 (in the post-IP) have been depleted after the immunoprecipitation with anti-CTPS2. The lower panel corresponds to the data normalized to the input as 100% of the three experiments. **(C)** Comparison of the levels of CTPS1 and CTPS2 in the immunoprecipitations with anti-CTPS2 (IP anti-CTPS2) normalized to the “input” as 100%. The lower panel corresponds to the ratio of CTPS2/CTPS1 in the IP anti-CTPS2 calculated from the upper panels showing 2.5–3.3-fold more CTPS2 than CTPS1 in the IP.

To further characterize the mechanisms of CTPS1-CTPS2 co-localization in cytoophidia, CTPS1-KO cells expressing GFP-CTPS1 or GFP alone were immunoprecipitated with anti-GFP–coupled beads and binding partners were identified by using quantitative label-free mass spectrometry. CTPS2 was found to be the main and most significant enriched protein associated with GFP-CTPS1. SUGP1, RTCB, and C14orf166 proteins were also found to be enriched in the GFP-CTPS1 pull-down (Fig 2A). SUGP1, RTCB, and C14orf166/RTRAF are involved in the processing of RNA. The specificity and the significance of these interactions are not known. However, CTPS2 and CTPS1 interaction was confirmed by co-immunoprecipitation experiments in WT HEK cells transiently transfected with GFP-tagged forms of CTPS1 and CTPS2. When immunoprecipitated, GFP-CTPS2 and GFP-CTPS1 pulled down endogenous CTPS1 and CTPS2, respectively (Figs 2B and S3A). No such interaction was detected in immunoprecipitates of the GFP alone. CTPS1 and CTPS2 were still able to bind with the GFP-CTPS2^H355A^ and GFP-CTPS1^H355A^ mutants, respectively, mutants that failed to form cytoophidia even upon treatment with 3-DU (Figs 2B and C and S3B). The CTPS1^H355A^ mutant was indeed previously reported to inhibit the tetramer–tetramer interface, disrupting polymer formation (Lynch et al, 2017). Therefore, these results suggest that the association between CTPS1 and CTPS2 is direct and occurs at the dimer or tetramer level, independently of polymers or cytoophidium formation. Association of WT endogenous forms of CTPS1 with CTPS2 was further confirmed and detected in control HEK cells when CTPS2 was immunoprecipitated (Fig 2D). Quantification of CTPS1 and CTPS2 in the input and post-IP lysates and in the anti-CTPS2 IP in WT HEK cells suggested that 17–27% of total CTPS1 are associated with CTPS2 in HEK cells with a CTPS2: CTPS1 ratio of 2.5–3 in the anti-CTPS2 immune complexes (Fig S4A–C).

### Both CTPS1 and CTPS1^H355A^ disrupt CTPS2 filamentous cytoophidia

**Figure 3.**
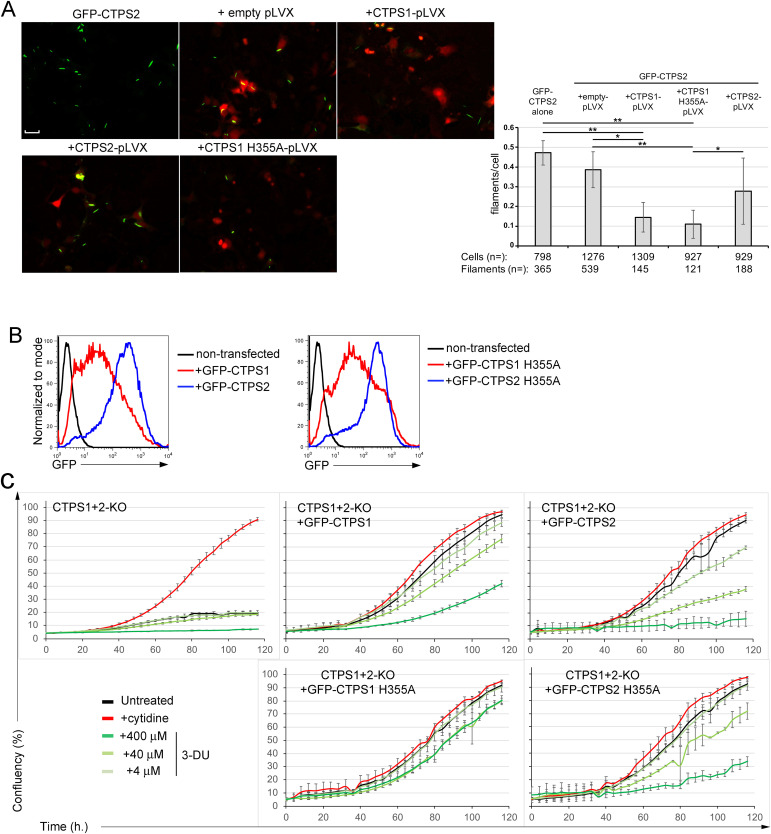
Disruption of CTPS2 cytoophidia by CTPS1 or CTPS1^H355A^ and effective proliferation of CTPS1+2-KO cells expressing CTPS1^H355A^ or CTPS2^H355A^. **(A)** CTPS1+2-KO HEK cells were transiently co-transfected with plasmids coding GFP-CTPS2 (in green) and pLVX vectors for CTPS1, CTPS2, or CTPS1^H355A^ (expressing mCherry as a reporter of transfection in red) in the absence of any treatment. Data from one representative experiment of two independent experiments. Bar graphs on the right panel correspond to the filament–cytoophidium/cell quantification from the images on the left with indicated numbers of cells and filaments–cytoophidia counted (n=) in three independent fields/images for each condition independently of the mCherry staining. Magnification 40x, scale bar: 25 μm. **(B, C)** CTPS1+2-KO cells stably expressing GFP-CTPS1, GFP-CTPS2, GFP-CTPS1^H355A^, or GFP-CTPS2^H355A^. Cells were then maintained in culture without cytidine. **(B)** Histograms of GFP expression of different cell lines. Data in the right and left panels are from the same experiment with the same “nontransfected” control histogram, which is representative of two independent experiments. **(C)** Confluency curves as percentages (%) showing the proliferation of different cell lines. Confluency was measured using an IncuCyte Zoom system. Cells were seeded for 24 h, then treated or not (untreated) with the indicated concentrations of 3-deazauridine (3-DU) or 200 μM cytidine. **(A)** Data as means ± SD. Mann–Whitney statistical tests, *P < 0.05 and **P < 0.01. n = is indicated in the panel.

**Figure S5.**
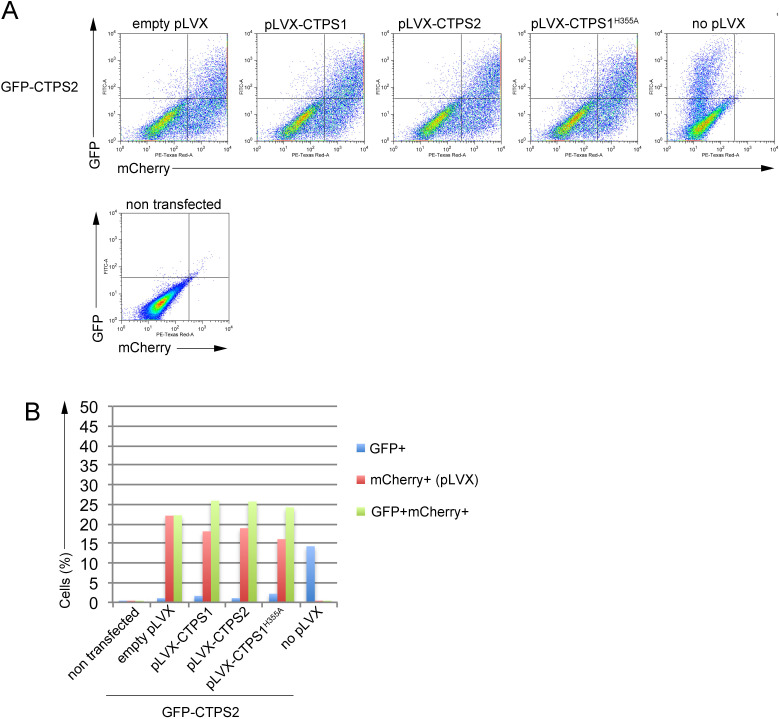
Control of GFP and mCherry expression of CTPS1+2-KO HEK cells transiently co-transfected with a plasmid coding GFP-CTPS2 and pLVX plasmids coding CTPS1, CTPS2, or CTPS1^H355A^ with the mCherry as a reporter gene (corresponding to Fig 3A). **(A)** FACS dot-plots of GFP and mCherry expression. **(A, B)** Bar graphs from flow-cytometry data as presented in (A), showing the percentage (%) of GFP+ alone, mCherry+ alone, and double-positive GFP+mCherry+cells.

Recent data have suggested that the CTPS1^H355A^ mutant could exert a dominant-negative effect on endogenous CTPS1 cytoophidia (Simonet et al, 2020; Chang et al, 2021). Furthermore, the filament assembly interface of CTPS2 tetramers was shown to be similar to that of CTPS1 (Lynch & Kollman, 2020). Thus, we hypothesized that the CTPS1^H355A^ mutant could also interfere with CTPS2 cytoophidium formation. To test this hypothesis, CTPS1^H355A^ or WT CTPS1 with mCherry reporter was transiently expressed together with GFP-CTPS2 in HEK CTPS1+2-KO cells (Figs 3A and S5A and B). We found that the expression of both the CTPS1^H355A^ mutant and WT CTPS1 disrupted both GFP-CTPS2 filaments, as shown by the decreased cytoophidia-shaped signal that correlated with an increased diffuse signal (Fig 3A). As CTPS1 and CTPS2 interact together independently of cytoophidia, one can speculate that WT CTPS1 or CTPS1^H355A^ when expressed might interfere with GFP-CTPS2 at the tetramer or dimer levels subsequently resulting in the block of cytoophidium formation. The observation that expression of WT CTPS1 also inhibited GFP-CTPS2 cytoophidia could be explained by the reduced intrinsic ability of CTPS1 to form cytoophidia compared with CTPS2 (as shown in Fig 2A) resulting in the inhibition of GFP-CTPS2 cytoophidia in this model.

### CTPS1 and CTPS2 are competent to promote proliferation independently of cytoophidia

**Figure S6.**
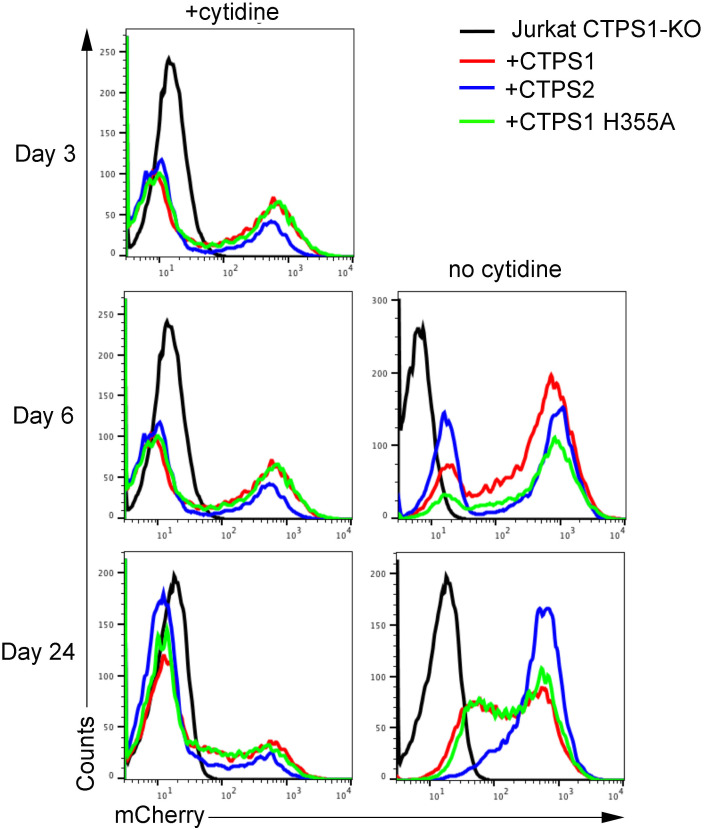
CTPS1^H355A^ is able to restore the proliferation of CTPS1-KO Jurkat cells as efficiently as WT CTPS1. Histograms of mCherry reporter expression profiles of CTPS1-KO Jurkat cells transduced with CTPS1 WT or CTPS1^H355A^ after or not cytidine deprivation (no cytidine) during 3, 6, and 24 d. The CTPS1 H355A profiles are similar to those of CTPS1 WT.

Taken together, these results suggest that the association between CTPS1 and CTPS2 at the molecular level is important for cytoophidium formation and its regulation. Recent studies have suggested that cytoophidia represent storage forms of CTPS1; however, it is not known what role these structures play in CTPS1- and CTPS2-dependent cell proliferation. Proliferation of CTPS1+2-KO HEK cells stably expressing GFP-CTPS1^H355A^ and GFP-CTPS2^H355A^ was compared with that of cells expressing GFP-CTPS1 or GFP-CTPS2 (Fig 3B). As seen with cells expressing GFP-CTPS2, cells reconstituted with CTPS2^H355A^ expressed higher levels of GFP (compared with cells expressing GFP-CTPS1^H355A^ or GFP-CTPS1). This indicates, as we previously reported, that CTPS2^H355A^, like WT CTPS2, has a lower capacity to promote proliferation associated with an increased sensitivity to 3-DU (compared with their CTPS1 counterparts), resulting in the selection of cells expressing higher levels of the enzyme (Minet et al, 2023). In the absence of cytidine, CTPS1+2-KO HEK cells expressing GFP-CTPS1^H355A^ and GFP-CTPS2^H355A^ proliferate with similar rates as cells expressing their GFP-tagged WT CTPS1 and CTPS2 counterparts. Of note, cells expressing GFP-CTPS1^H355A^ and GFP-CTPS2^H355A^ mutants were more resistant to inhibition by 3-DU compared with their WT counterparts that could suggest an enhanced activity associated with the H355A mutation (Fig 3C). Supplementation with cytidine did not change or only slightly affected proliferation rates. In contrast, CTPS1+2 KO cells alone or reconstituted with GFP alone failed to proliferate in the absence of cytidine supplementation. Finally, a nontagged form of CTPS1^H355A^ was able to restore the proliferation of CTPS1-KO Jurkat cells as efficiently as WT CTPS1 (Fig S6). Of note, Jurkat cells are highly dependent on CTP and CTPS1 for their rapid proliferation (Minet et al, 2023). Therefore, these results indicate that cytoophidium formation is not necessary for GFP-CTPS1 and GFP-CTPS2 to promote proliferation of HEK cells.

### The interaction between CTPS1 and CTPS2 modulates their activity and regulation

**Figure 4.**
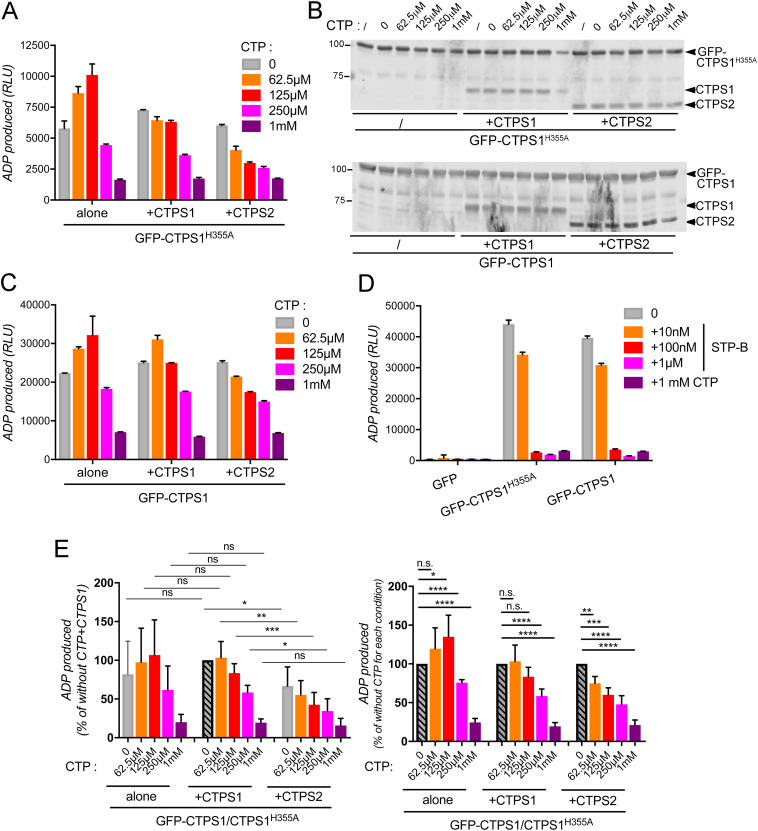
Association between CTPS1 and CTPS2 regulates the enzymatic activity of the complex. **(A)** On-beads enzymatic assay for CTPS activity, after immunoprecipitation (anti-GFP–coated beads) of GFP-CTPS1^H355A^ in the presence or not of WT CTPS1 or CTPS2, measured by the synthesis of ADP in the presence of UTP and various concentrations of CTP. **(B)** Western blots (WB) of immunoprecipitates of GFP-CTPS1^H355A^ (upper panel) or GFP-CTPS1 (lower panel) corresponding to the experiment in (A) and in (C), respectively. WB with anti-GFP, anti-CTPS1, and anti-CTPS2 antibodies showing the association between GFP-CTPS1^H355A^ and CTPS1 or CTPS2 that validates the homogeneity of the protein levels on the beads used for the enzymatic reaction. One representative experiment of three with three biological replicates. **(C)** Same as in (A), except that a GFP-WT CTPS1 was used (GFP-CTPS1) in place of a GFP-CTPS1^H355A^. One representative experiment of two with three biological replicates. **(D)** Same as in (A, B, C) except that the on-beads CTPS enzymatic assay of GFP-CTPS1 or GFP-CTPS1^H355A^ alone was assessed in the presence of increasing concentrations of STP-B, a selective inhibitor of CTPS1 or 1 mM of CTP. One representative experiment of two with three biological replicates. Data as means ± SD. **(E)** Same as in (A, B, C). Data are normalized from five independent experiments (n = 5) including two experiments with the GFP-CTPS1 and three experiments with the GFP-CTPS1^H355A^. The conditions representing 100% are depicted in gray with black zebra stripes. Unpaired and paired *t* tests, *P < 0.05; **P < 0.01; ***P < 0.001; ****P < 0.0001; ns, nonsignificant. **(A, C, D, E)** Data as means ± SD.

CTPS1 and CTPS2 are co-expressed in most tissues. Despite their high homology, an array of data suggests that the activity levels of CTPS1 and CTPS2 (Lynch et al, 2021; Minet et al, 2023) and their regulation (Lynch & Kollman, 2020; Lynch et al, 2021) are different. In particular, human CTPS2 is less active and more sensitive to CTP negative feedback and to the inhibition by UTP analog, 3-deazauridine (Lynch et al, 2021; Minet et al, 2023). To further explore the potential functional consequences of the interaction between CTPS1 and CTPS2 in the absence of cytoophidium formation, we analyzed the CTPS activity of the GFP-CTPS1^H355A^ when associated with untagged CTPS1 or CTPS2. GFP-CTPS1^H355A^ prevented cytoophidium formation and also allowed a better protein recovery in immunoprecipitation experiments. For this purpose, lysates containing GFP-CTPS1^H355A^ were mixed with lysates containing either nontagged CTPS1 or CTPS2, obtained from CTPS1+2-KO HEK cells transiently overexpressing GFP-CTPS1^H355A^, CTPS1 or CTPS2. GFP-CTPS1^H355A^ was then immunoprecipitated using anti-GFP beads, and the CTPS enzymatic activity on beads was monitored in the presence of UTP and ATP as substrates and increased concentrations of CTP, by quantification of ADP produced during the enzymatic reaction (Fig 4A) as previously reported (Novak et al, 2022; Minet et al, 2023). The presence of nontagged CTPS1 or CTPS2 in GFP-CTPS1^H355A^ complexes was verified by Western blotting of the immunoprecipitated proteins (from anti-GFP beads) at the end of the enzymatic reaction, confirming the association of CTPS1 or CTPS2 with GFP-CTPS1^H355A^ (Fig 4B, upper panel). Of note, the addition of lysates containing CTPS1 or CTPS2 did not affect the level of immunoprecipitation of GFP-CTPS1^H355A^.

GFP-CTPS1^H355A^ activity alone in the absence of CTPS1 or CTPS2 was increased by the addition of low concentrations of CTP (62.5 and 125 μM) and decreased with high concentrations of CTP (250 μM and 1 mM) (Fig 4A). This increased activity at low doses of CTP might reflect a cooperative effect of low doses of CTP promoting the assembly of tetramers from dimers (at dilute enzyme concentrations), whereas the decreased activity is dependent on a negative feedback (McPartland & Weinfeld, 1979). Association of CTPS1 proteins with GFP-CTPS1^H355A^ reduced or abolished the positive effects of low doses of CTP. Notably, this was further exacerbated when CTPS2 was associated (with GFP-CTPS1^H355A^) resulting in a global decrease in the activity at all CTP concentrations except the highest (CTP 1 mM). Similar effects were observed if the GFP-CTPS1^H355A^ was replaced by a GFP-WT CTPS1 protein suggesting the cytoophidium formation did not interfere with these assays (Fig 4B and C, lower panel). The increased activity associated with GFP-CTPS1 and GFP-CTPS1^H355A^ alone at low doses of CTP, that is not observed when CTPS1 was added, might be explained by modification in the sensitivity to CTP of CTPS1 when tagged with the GFP. This cooperative effect might reflect the presence of destabilized tetramers associated with the GFP-tagged forms (but not with nontagged CTPS1) that are stabilized by low CTP concentrations (Levitzki & Koshland, 1972). Furthermore, ADP production in these conditions was selectively dependent on the CTPS activity as ADP production was fully blocked by the addition of STP-B, a chemical inhibitor specific of CTPS1 (Asnagli et al, 2023; Durand et al, 2024; Pfeiffer et al, 2024), suggesting that no other ATPase activity (that could have co-immunoprecipitated with the anti-GFP beads) accounted for ADP production (Fig 4D). Normalization and consolidation of the experiments showed in Fig 4A and C confirmed these results, showing that the presence of CTPS2 in GFP-CTPS1/CTPS1^H355A^ complexes (in comparison with the presence of CTPS1) significantly decreased the CTPS activity of the complexes (Fig 4E, left panel) and increased their sensitivity to CTP feedback inhibition at low doses of CTP (Fig 4E, right panel). Therefore, this indicates that the association between CTPS2 and CTPS1 can modulate CTPS1 activity.

## Discussion

In our study, we show that CTPS1 and CTPS2 directly and physically interact together. This interaction likely occurs at the tetramer level as it does not require cytoophidium formation, tetramer being considered as the active form of the enzyme. The interaction between CTPS1 and CTPS2 may result in cooperative and regulatory effects on CTPS activity as hinted by our observations. Importantly, this association represents a novel level of regulation of the CTPS activity through CTPS1 and CTPS2 interaction as previously suggested (Lynch & Kollman, 2020).

We found that the requirements to form these large filament structures termed cytoophidia differ between CTPS1 and CTPS2 in HEK cells. We showed that 3-DU–induced endogenous CTPS2 cytoophidia is dependent upon the presence of CTPS1 that probably co-localizes in the cytoophidia. In the absence of CTPS1, we failed to induce CTPS2 cytoophidia with 3-DU. This appears rather surprising because CTPS2 is more sensitive to 3-DU in terms of inhibition of enzymatic activity and CTPS2-dependent proliferation. Expression levels of CTPS1 and CTPS2 in HEK cells are similar and the absence of CTPS1 did not alter CTPS2 expression (Minet et al, 2023), thus likely excluding that lack of CTPS2 cytoophidia (in the absence of CTPS1) results from lower or insufficient expression of CTPS2. In addition, it has been suggested that the filament assembly interfaces are less stable in human CTPS1 than in CTPS2 (Lynch & Kollman, 2020), which should be in favor of CTPS2 cytoophidium formation by itself independently of CTPS1. However, this dependency of CTPS2 cytoophidium formation on CTPS1 strongly supports that endogenous CTPS1 and CTPS2 co-localize in the same cytoophidia in cells co-expressing CTPS1 and CTPS2. Our results also suggest that the requirements for CTPS2 cytoophidia are different than those of CTPS1 (as CTPS2 alone in contrast to CTPS1 was not able to form detectable cytoophidia that depended upon CTPS1 in our experimental conditions). Unfortunately, for technical issues, we were not able to directly test the co-localization of endogenous CTPS1 and CTPS2 in filaments (as both our anti-CTPS1 and CTPS2 antibodies were from the same species and not directly coupled to fluorochromes).

Using GFP-tagged forms, we observed that both CTPS1 and CTPS2 spontaneously form cytoophidia, but in contrast to GFP-CTPS1 cytoophidia, GFP-CTPS2 cannot be induced to further form cytoophidia in the presence of CTP or 3-DU. Interestingly, Lynch and Kollman showed that UTP/substrate-bound and CTP/product-bound tetramers of purified CTPS2 can form polymers in vitro (Lynch & Kollman, 2020). In particular, they observed using a CTPS2 mutant forming spontaneously covalently linked polymers (that cannot dissociate), that CTP could still access its binding pocket in filament polymer form and modulate the conformation of the tetramers, showing that the product/substrate balance can dynamically switch the conformation of the CTPS2 tetramer between its substrate-bound and product-bound states without requiring prior polymer dissociation. This might explain the absence of detectable changes (by microscopy) in GFP-CTPS2 cytoophidia, once formed (and potentially already bound to CTP and/or UTP) in the presence of cytidine or 3-DU. However, it should be pointed out that these conditions used to induce cytoophidium formation remain artificial because they may be influenced or dependent on the ability of the GFP to form low-affinity dimers (Chang et al, 2018); thus, the interpretation of these data must be cautious. However, GFP dimerization is insufficient to induce cytoophidia as cytoophidia are lost with the H355A mutation in both CTPS1 and CTPS2. Moreover, it is not clear yet what is the physiological role of these CTPS1/2 cytoophidia in vivo. Naturally occurring CTPS cytoophidia have been observed in cancer tissues (Chang et al, 2017). In any case, our observations suggest that in addition to differences in their enzymatic activity, CTPS1 and CTPS2 also differ in their requirements for cytoophidium formation.

Several interesting observations came out from the study of the H355A mutation that completely abolishes the ability of CTPS1 and CTPS2 to form polymers (Lynch et al, 2017; Lynch & Kollman, 2020). Notably, the CTPS1^H355A^ and CTPS2^H355A^ mutants were still able to interact with endogenous CTPS2 or CTPS1, respectively, suggesting that the CTPS1-CTPS2 interaction occurs at the tetramer level and does not require polymer formation. However, this interaction is required for cytoophidium formation as highlighted by the observation that the overexpression of both CTPS1^H355A^ mutant and CTPS1 blocks GFP-CTPS2 cytoophidium formation. Another interesting finding is the capacity of GFP-CTPS1^H355A^ and GFP-CTPS2^H355A^ mutants to promote the proliferation of CTPS1/CTPS2-deficient cells similarly to wild-type CTPS1 and CTPS2 GFP forms, suggesting proliferation and cytoophidium formation are disconnected and cytoophidium formation is not required for cell proliferation (at least in the cell model studied here). However, we cannot exclude an effect of the GFP tag, although unlikely, because we did not have evidence that its addition modified the function of CTPS1/2 in the different experiments conducted here and in our previous studies (Minet et al, 2023).

We have examined the functional impact of the interaction between CTPS1 and CTPS2 on CTPS enzymatic activity from lysates produced in HEK cells. We observe that the presence of CTPS2 makes CTP synthesis more sensitive to the product feedback regulation. We cannot exclude that CTPS2 natural partners (in the lysates) present in the reactions possibly further modulate the CTPS activity. At this stage, the stoichiometry of association between CTPS1 and CTPS2 is not known. Variations in the balance between CTPS1 and CTPS2 expression in the cell may in turn lead to different association ratios inside the tetramer, potentially modifying the activity and regulation of the resulting structure. We previously observed in the HEK cells (in which both CTPS1 and CTPS2 are equally expressed) that the inactivation of CTPS2 (CTPS2 KO cells) had no detectable effect on the global CTP synthase activity of these cells. However, in the absence of CTPS1 (CTPS1 KO cells), CTPS2 alone was able to provide a sufficient (but decreased) CTP synthase activity to maintain cell proliferation (although the proliferation rate was diminished) (Minet et al, 2023). Because CTPS2 is more sensitive to the CTP negative feedback (Lynch et al, 2021), CTP levels in wild-type HEK cells (in the presence of CTPS1) may be sufficient to inhibit CTPS2 activity, thus explaining why in absence of CTPS2, the global CTPS activity remains unchanged. We observed that several cancer cell lines of T-cell lymphoid origin did not express CTPS2 such as Jurkat, MOLT-4, and HUT-78 (Minet et al, 2023). It would be interesting to explore the events that led to the loss or absence of CTPS2 expression and to identify other cell lines that may have decreased to absent levels of CTPS2. In light of our results, we can hypothesize that the interaction between CTPS1 and CTPS2 may modulate their activity and cell proliferation. The loss of one of the enzymes would remove this layer of regulation, leading to changes in the enzymatic activity and its regulation. Our results suggest that the association of CTPS2 with CTPS1 lowers the enzymatic activity of the complex and its sensitivity to the inhibitory feedback loop by low CTP concentrations. Thus, one can hypothesize that the decreased or absence of CTPS2 expression could benefit tumor cells in particular T-cell lines providing a selective advantage. Additional experiments are warranted to test this possibility.

In conclusion, our study identifies a novel regulatory mechanism of the CTP synthetase activity resulting from the interaction between CTPS1 and CTPS2. This may have important consequences for the therapeutic use of CTPS1 inhibitors. Indeed, specific CTPS1 inhibitors may have their efficiency modulated by the presence of CTPS2, not only because of a compensatory effect of CTPS2 on the global CTP synthase activity level, but also because of the consequences of an interaction with CTPS2. Potentially, this could for instance result in a decreased inhibitory activity on CTPS1 or in an indirect effect on CTPS2 through its interaction with CTPS1.

## Materials and Methods

### Plasmids

The plasmids containing cDNAs coding full-length CTPS1 and CTPS2 were obtained by PCR as previously described (Martin et al, 2014; Minet et al, 2023) using Q5 High-Fidelity DNA Polymerase (New England Biolabs). The cDNAs were verified by sequencing and inserted into a bicistronic lentiviral expression vector encoding mCherry as a reporter (pLVX-EF1α-IRES-mCherry Vector; Clontech). For GFP tagging, the cDNAs were inserted into a pEGFP-C1 vector (Clontech). All constructs were validated by Sanger sequencing using BigDye Terminator v3.1 Cycle Sequencing Kit (Life Technologies) and 3500xL Genetic Analyzer (Applied Biosystems) according to the manufacturer’s instructions. Sequence analysis was performed using DNADynamo (BlueTractorSoftware).

### Site-directed mutagenesis

Site-directed mutagenesis was performed using Q5 Site-Directed Mutagenesis Kit (New England Biolabs) according to the manufacturer’s instructions using the following primers: CTPS1^H355A^ F: gccgaagcTTGGCAGAAGCTCTGTAGTG/R: gta​gcg​cac​GGG​CTC​TTC​TTG​CGA​GGT and CTPS2^H355A^ F: tgaagcttggcAGAAGCTATGCAAAGCTGATG/R: gca​aat​ttc​aca​GGG​TCC​TCG​GTT​TCA​GTG, and validated by Sanger sequencing.

### Cell culture

HEK293T cells (ATCC) were cultured in DMEM, 10% FBS, and 1% PS (complete DMEM). Calcium- and magnesium-free PBS and 1x trypsin–EDTA were respectively used to wash the cells and to detach adherent cells. All of the abovementioned reagents were from Life Technologies. CTPS1- and/or CTPS2-deficient cell lines were maintained with 200 μM of cytidine (Sigma-Aldrich). 3-Deazauridine (3-DU) (Sigma-Aldrich) was used as a nonselective inhibitor of CTPS1 and CTPS2.

### Cell transfection

HEK293T cells were transfected by lipofection with Lipofectamine 3000 (Life Technologies) according to the manufacturer’s instructions.

### CTPS1-KO cell line generation

CTPS1-KO, CTPS2-KO, and CTPS1+2-KO cell lines were generated as described previously using the CRISPR-Cas9 technology (Minet et al, 2023). The expression of CTPS1, CTPS2, and/or GFP has been verified regularly before use of the cell lines by Western blotting and remained stable over time.

### Cell proliferation

Proliferation of HEK cells was analyzed as a percentage of confluency. For that, cells were seeded in complete culture medium at a density of 6,250 cells/cm^2^ for 24 h and placed in an IncuCyte Zoom Live-Cell analysis system (Sartorius). Phase-contrast and GFP fluorescence pictures were taken every 3 h and analyzed using a confluency mask.

### Cytometry

All data were collected on an LSRFortessa cytometer and analyzed using FlowJo version 9.3.2 software (BD Biosciences).

### Immunocytochemistry

HEK cells were cultured on presterilized, polylysine-coated (Sigma-Aldrich) glass slides in 24-well plates in complete DMEM for 24 h, then treated as indicated. The glass slides were then removed from the wells, fixed for 15 min with 4% paraformaldehyde in PBS (Sigma-Aldrich), neutralized with 0.1 M glycine (Life Technologies) for 2 min, and permeabilized using 0.5% Triton X-100 (Sigma-Aldrich) for 15 min. The cells were saturated, stained with rabbit monoclonal anti-CTPS1 EPR8086 (ab133743; Abcam) or rabbit polyclonal anti-CTPS2 (C-ter) (ab190462; Abcam) antibodies, and then stained with AF488- or AF346-conjugated goat anti-rabbit antibodies (Life Technologies) in PBS, BSA 1% for 1 h each and then with 1 μg/ml DAPI for 5 min. Transfected and glass-mounted cell images were captured using Axio Vert.A1 Inverted Microscope (Zeiss) unless specified otherwise. Filament–cytoophidia and DAPI-stained nucleus quantifications were performed using ImageJ.

### Western blot

Cell lysates were prepared and analyzed using standard procedures as previously described (Martin et al, 2014, 2020). The following antibodies were used for immunoblotting: rabbit polyclonal anti-actin (A2066; Sigma-Aldrich), rabbit monoclonal anti-CTPS1 EPR 8086 (ab133743; Abcam), rabbit polyclonal anti-CTPS2 (C-ter) (ab190462; Abcam), rabbit anti-GFP (2956S; Cell Signaling). The following antibodies were used for detection: IRDye 680RD- or IRDye 800CW-conjugated goat anti-rabbit or goat anti-mouse (925-68071, 925-32211, 925-68070, and 925-32210; LI-COR), and membranes were analyzed with the Odyssey CLX imager (LI-COR) and quantified using Image Studio Lite 5.5.

### Immunoprecipitation

For CTPS2 immunoprecipitation, cells were lysed in complete TNE buffer (NaF 1 mM, Tris 50 mM, pH 8, NP-40 1%, EDTA 20 mM) containing protease inhibitor cocktail (#11836145001; Roche) and phosphatase inhibitors II and III (#P5726 and #P0044; Sigma-Aldrich) as previously described (Martin et al, 2020). Lysates were precleared using 15 μl of Protein A-Sepharose beads (Cytiva) for 40 min. 4 μg of rabbit polyclonal anti-CTPS2 (C-ter) (ab190462; Abcam) antibody was added to the cleared lysates for 90 min before the addition for 1 h of 15 μl of Protein A-Sepharose beads. Beads were then washed five times in lysis buffer and diluted in sample buffer, incubated for 10 min at room temperature, then boiled for 5 min, and analyzed by SDS–PAGE and Western blot.

### Affinity purification–mass spectrometry

Cell lysates of CTPS1-KO cells stably expressing GFP alone or GFP-CTPS1 were obtained by incubating 30 × 10^6^ cells in a lysis buffer containing 20 mM Tris–HCL, pH 7.5, 1 mM NA_3_VO_4_, 1 mM NaF, 1% NP-40, 1 mM PMSF, NaCl 300 mM, and EDTA-free protease inhibitors (#11836145001; Roche) for 30 min at 4°C, adjusted to 150 mM NaCl, then centrifuged for 30 min at 4°C. GFP immunoprecipitation from cell lysates was performed using GFP-Trap agarose beads (ChromoTek) for 90 min. The beads were then washed five times in lysis buffer and either submitted for proteomics analyses or diluted in sample buffer, boiled for 5 min, and analyzed by SDS–PAGE and Western blot. For proteomics analyses, anti-GFP beads were washed thrice with 25 mM NH4HCO3 and resuspended in 100 μl of 25 mM NH_4_HCO_3_ and digested by adding 0.2 μg of trypsin/LysC (Promega) for 1 h at 37°C which allows to shave the surface proteins without going to the core of the antibody. Samples were then loaded into custom-made C18 StageTips packed by stacking one AttractSPE disk (#SPE-Disks-Bio-C18-100.47.20; Affinisep) and 2 mg beads (#186004521 Sep-Pak C18 Cartridge; Waters) into a 200-μl micropipette tip for desalting. Peptides were eluted using a ratio of 40:60 MeCN:H_2_O + 0.1% formic acid and vacuum-concentrated to dryness. Peptides were reconstituted in injection buffer (0.3% TFA) before liquid chromatography–tandem mass spectrometry (LC-MS/MS) analysis. Online chromatography was performed using an RSLCnano system (Ultimate 3000; Thermo Fisher Scientific) coupled to an Orbitrap Fusion Tribrid mass spectrometer (Thermo Fisher Scientific). Peptides were trapped in a C18 column (75 μm inner diameter × 2 cm; nanoViper Acclaim PepMap 100; Thermo Fisher Scientific) with buffer A (2:98 MeCN:H2O in 0.1% formic acid) at a flow rate of 4.0 μl/min over 4 min. Separation was performed on a 50 cm × 75 μm C18 column (nanoViper Acclaim PepMap RSLC, 2 μm, 100 Å; Thermo Fisher Scientific), regulated to a temperature of 55°C with a linear gradient of 5–25% buffer B (100% MeCN in 0.1% formic acid) at a flow rate of 300 nl/min over 100 min. Full-scan MS was acquired using an Orbitrap Analyzer with the resolution set to 120,000, and ions from each full scan were fragmented with higher energy C-trap dissociation (HCD) and analyzed in the linear ion trap in rapid mode. Normalized collision energy was set to 30, automatic gain control target to 20,000, maximum injection time to 100 ms, and dynamic exclusion to 30 s. For peptide identification, the data were searched against the *Homo sapiens* UP000005640 database using Sequest HT through Proteome Discoverer (v.2.2). Enzyme specificity was set to trypsin, and a maximum of two missed cleavage sites were allowed. Oxidized methionine and N-terminal acetylation were set as variable modifications. Maximum allowed mass deviation was set to 10 ppm for monoisotopic precursor ions and 0.6 Da for MS/MS peaks. The resulting files were further processed using myProMS (Poullet et al, 2007) v.3.9.3 (work in progress). False discovery rate (FDR) was calculated using Percolator (The et al, 2016) and was set to 1% at the peptide level for the whole study. Label-free quantification was performed using peptide extracted ion chromatograms (XICs), computed with MassChroQ (Valot et al, 2011) v.2.2.1. For protein quantification, XICs from proteotypic peptides shared between compared conditions (TopN matching) were used. Missed cleavages were not allowed for quantification. Median and scale normalization at the peptide level was applied to the total signal to correct the XICs for each biological replicate (N = 4). To estimate the significance of the change in protein abundance, a linear model (adjusted on peptides and biological replicates) was performed, and *P*-values were adjusted using the Benjamini–Hochberg FDR procedure. To eliminate nonspecifically isolated proteins, only proteins with at least three total peptides in all replicates, a twofold enrichment, and an adjusted *P* ≤ 0.05, and for the unique proteins three total peptides, in CTPS1-KO+GFP-CTPS1 samples versus control GFP samples were selected. In all replicates of CTPS1-KO+GFP-CTPS1 or CTPS1-KO+GFP alone samples, more than 20 peptides corresponding to the GFP were identified representing a >20-fold increase compared with samples of nontransfected CTPS1-KO HEK cells. The mass spectrometry proteomics data have been deposited to the ProteomeXchange Consortium (http://proteomecentral.proteomexchange.org) via the PRIDE (Perez-Riverol et al, 2022) partner repository with the dataset identifier PXD047654. The following samples were used for analysis: for the GFP alone group: C9468VM, C9471VM, C9474VM, C9477VM; and for the GFP-CTPS1 group: C9469VM, C9472VM, C9475VM, and C9478VM.

### CTPS activity on immunoprecipitated enzymes

GFP-tagged H355A CTPS1 or GFP-tagged WT CTPS1 in C1 EGFP vectors and WT nontagged CTPS1 and CTPS2 in pLVX-EF1a-IRES-mCherry vectors were transiently overexpressed in HEK cells deficient for CTPS1 and CTPS2 (CTPS1+2 KO cells) expression using Lipofectamine 3000 (Life Technologies). 48 h post-transfection, cells were harvested by trypsinization and lysed in a lysis buffer (TNE) containing 50 mM Tris, 150 mM NaCl, 10 mM EDTA, 1% NP-40 complemented with phosphatase inhibitors 2 and 3 (Sigma-Aldrich), cOmplete protease inhibitor tablets (Roche), and 25 mM NaF (Sigma-Aldrich). Lysates were cleared of debris by centrifugation. To associate the two enzymes, lysates containing GFP-tagged CTPS1 were mixed 50:50 with TNE alone or TNE lysates of double KO HEK overexpressing WT CTPS1 or CTPS2 and agitated on a rotating tube mixer for 60 min at 4°C. Immunoprecipitation was performed on at least 3 mg of proteins on a rotating tube mixer for 1 h at 4°C using 24 μl of GFP-Trap agarose beads (ChromoTek) equilibrated in TNE buffer. Beads were then washed three times in TNE buffer, then three times in 70 mM Hepes, pH = 8.0, and 4 μl of beads was used for each enzymatic reaction. Enzymatic reactions were performed in 70 mM Hepes, pH 8.0, containing 1,7 mM EDTA, 13 mM MgCl2, 1,3 mM ATP, 0,2 mM GTP (Life Technologies), 13 mM glutamine, 10 mM NaF (Sigma-Aldrich) in the presence of 1,3 mM UTP and the indicated doses of CTP (Life Technologies) or STP-B, a selective inhibitor of CTPS1 (Durand et al, 2024; Pfeiffer et al, 2024) for 120 min at 37°C. The enzymatic activity was assessed by quantifying the ADP produced in the supernatant during the enzymatic reaction using the ADP-Glo Max kit (Promega) in 384-well plates according to the manufacturer’s instructions. After the enzymatic reactions, the beads were resuspended in 2x Laemmli buffer, boiled for 5 min at 100°C, and analyzed by SDS–PAGE followed by Western blotting against GFP (mouse monoclonal anti-GFP (Life Technologies); rabbit monoclonal anti-CTPS1 and polyclonal anti-CTPS2 (C-ter) (Abcam)).

## Supplementary Material

Reviewer comments

## Data Availability

Datasets of mass spectrometry and proteomics are publicly available on the PRIDE repository under the number PXD047654 referred to as Identification of CTPS1 partners.
